# Nuclear, Cytosolic, and Surface-Localized Poly(A)-Binding Proteins of *Plasmodium yoelii*

**DOI:** 10.1128/mSphere.00435-17

**Published:** 2018-01-10

**Authors:** Allen M. Minns, Kevin J. Hart, Suriyasri Subramanian, Susan Hafenstein, Scott E. Lindner

**Affiliations:** a Department of Biochemistry and Molecular Biology, Pennsylvania State University, University Park, Pennsylvania, USA; b Center for Malaria Research, Pennsylvania State University, University Park, Pennsylvania, USA; c Department of Medicine, Pennsylvania State University, College of Medicine, Hershey, Pennsylvania, USA; Indiana University School of Medicine

**Keywords:** PABP, *Plasmodium*, RNA metabolism, electron microscopy, poly(A)-binding protein, surface proteins, *yoelii*

## Abstract

Malaria is a devastating illness that causes approximately 500,000 deaths annually. The malaria-causing parasite (*Plasmodium* genus) uses the process of translational repression to regulate its growth, development, and transmission. As poly(A)-binding proteins (PABP) have been identified as critical components of RNA metabolism and translational repression in model eukaryotes and in *Plasmodium*, we have identified and investigated two PABPs in *Plasmodium yoelii*, PyPABP1 and PyPABP2. In contrast to most single-celled eukaryotes, *Plasmodium* closely resembles metazoans and encodes both a nuclear PABP and a cytosolic PABP; here, we provide multiple lines of evidence in support of this observation. The conserved domain architectures of PyPABP1 and PyPABP2 resemble those of yeast and metazoans, while multiple independent binding assays demonstrated their ability to bind very strongly and specifically to poly(A) sequences. Interestingly, we also observed that purified PyPABP1 forms homopolymeric chains despite exhaustive RNase treatment *in vitro*. Finally, we show by indirect immunofluorescence assays (IFAs) that PyPABP1 and PyPABP2 are cytoplasm- and nucleus-associated PABPs during the blood stages of the life cycle. Surprisingly, however, PyPABP1 was instead observed to also be localized on the surface of transmitted salivary gland sporozoites and to be deposited in trails when parasites glide on a substrate. This is the third RNA-binding protein verified to be found on the sporozoite surface, and the data may point to an unappreciated RNA-centered interface between the host and parasite.

**IMPORTANCE** Malaria remains one of the great global health problems. The parasite that causes malaria (*Plasmodium* genus) relies upon exquisite control of its transmission between vertebrate hosts and mosquitoes. One crucial way that it does so is by proactively producing mRNAs needed to establish the new infection but by silencing and storing them until they are needed. One key protein in this process of translational repression in model eukaryotes is poly(A)-binding protein (PABP). Here we have shown that *Plasmodium yoelii* utilizes both a nuclear PABP and a cytosolic PABP, both of which bind specifically to polyadenylated RNA sequences. Moreover, we find that the cytosolic PABP forms chains *in vitro*, consistent with its appreciated role in coating the poly(A) tails of mRNA. Finally, we have also verified that, surprisingly, the cytosolic PABP is found on the surface of *Plasmodium* sporozoites. Taking the data together, we propose that *Plasmodium* utilizes a more metazoan-like strategy for RNA metabolism using specialized PABPs.

## Abstract (precis)

Malaria remains one of the great global health problems. The parasite that causes malaria (*Plasmodium* genus) relies upon exquisite control of its transmission between vertebrate hosts and mosquitoes. One crucial way that it does so is by proactively producing mRNAs needed to establish the new infection but by silencing and storing them until they are needed. One key protein in this process of translational repression in model eukaryotes is poly(A)-binding protein (PABP). Here we have shown that *Plasmodium yoelii* utilizes both a nuclear PABP and a cytosolic PABP, both of which bind specifically to polyadenylated RNA sequences. Moreover, we find that the cytosolic PABP forms chains *in vitro*, consistent with its appreciated role in coating the poly(A) tails of mRNA. Finally, we have also verified that, surprisingly, the cytosolic PABP is found on the surface of *Plasmodium* sporozoites. Taking the data together, we propose that *Plasmodium* utilizes a more metazoan-like strategy for RNA metabolism using specialized PABPs.

## INTRODUCTION

Malaria is a global health problem that continues to affect around 250 million people annually, resulting in 250,000 to 650,000 deaths (1). Because of this, great effort is being expended to understand how the malaria-causing parasite (*Plasmodium* genus) survives and regulates its lifecycle within the vertebrate host and mosquito vector. *Plasmodium* species have a complex lifecycle as they grow and develop in both female *Anopheles* mosquitos and vertebrate hosts, a process that requires extensive regulatory control. When a mosquito carrying sporozoites in its salivary glands takes a blood meal, the sporozoites are injected into the skin of the host, enter the bloodstream, and infect hepatocytes. Within hepatocytes, the parasites develop into tens of thousands of merozoites, which are released into the bloodstream and invade red blood cells. It is only during this erythrocytic stage that the malarial symptoms present. The sexual forms of the parasite (male and female gametocytes) begin to develop later in the erythrocytic stage, which can productively infect mosquitoes following a new blood meal. These parasites develop in the midgut of the mosquito for approximately 2 weeks and, as sporozoites, enter the salivary gland and effectively start the lifecycle over again (2).

Posttranscriptional regulation is an essential process that the parasite uses to regulate its gene expression and life cycle progression. For instance, the gametocyte and sporozoite transmission stages must remain stable and infectious in the host or vector (respectively) for long periods of time before a mosquito may bite. At these stages, a prevailing model states that transcripts that are important for the subsequent infection of the host or vector are translationally repressed so that protein synthesis can occur rapidly, presumably when the opportunity to infect arises (3). These translationally repressed transcripts are stored in punctate storage granules within the cytoplasm, as also occurs in model eukaryotes (4). Several proteins, including DOZI, CITH, ALBA proteins, PUF proteins, and the CAF1/CCR4/NOT complex, which are involved in gene regulation through translational repression, can be found interacting with transcripts within these granules (5–14). DOZI is an RNA helicase that has been shown to be involved in translational repression and maintenance of the gametocyte-specific transcripts that are used to synthesize proteins that are important in zygote development. The loss of DOZI still allows the gametes to fuse, but they are unable to continue meiotic replication, resulting in the inability of the zygote to develop into the ookinete (13, 14). CITH, a verified binding partner of DOZI, has also been shown to interact with translationally repressed transcripts important for zygote development, and its genetic deletion causes complete arrest (13). These cytosolic granules also contain proteins important for regulating gene expression during blood stage infection. For instance, ALBA proteins such as *Plasmodium falciparum* ALBA1 (PfALBA1), which is an essential regulator of translation during blood stage infection, have been shown to be involved in translational repression (7). ALBA proteins such as *Plasmodium yoelii* ALBA4 (PyALBA4), which has been shown to be involved in regulating male gametocyte activation and synchronous sporozoite development, are also important in other parts of the life cycle as well. When PyALBA4 was genetically deleted, there was an increase in the number of activated male gametocytes and a loss of the coordinated egress of sporozoites from the midgut (5). The CAF1/CCR4/NOT complex exists in cytosolic granules that regulate RNA degradation, translation, and other critical RNA-centered processes in model eukaryotes (12). Lastly, PUF proteins have also been shown to be involved in gene regulation through translational repression in the transmitted parasite stages. For instance, Puf2 in *P. falciparum* interacts directly with translationally repressed transcripts that encode proteins that are involved in gametocytogenesis. When PfPuf2 is deleted, there is premature expression of these proteins (15).

What unites all of these translationally repressive proteins and their complexes in model eukaryotes is that they all interact with and are influenced by poly(A)-binding proteins (PABPs). PABPs are essential proteins involved in everything from the addition/removal of poly(A) sequences on the 3′ end of transcripts to RNA stability and translational control (16) (reviewed in reference 17). However, the precise functions of PABP in these discrete storage granules in *Plasmodium* are understudied and underappreciated.

In model eukaryotes, single-celled organisms typically encode a single, cytosolic PABP whereas metazoans encode at least two (nuclear and cytosolic) PABPs. For instance, humans encode PABPC1 for predominantly cytosolic functions and PABPN1 for maturation and export of mRNAs from the nucleus (reviewed in reference 16). PABPN1 functions in the nucleus to stimulate 3′-poly(A) tail addition through its interaction with poly(A) polymerase (PAP) and protects the growing tail from degradation. PABPN1 also regulates the final length of the 3′-poly(A) tail and protects it before export from the nucleus (18, 19). PABPC1, which is primarily cytosolic, can enter the nucleus to retrieve mRNA and replace PABPN1 during mRNA export from the nucleus (16). Lastly, a closed-loop model of mRNA invokes the interaction of PABPC1 [bound to the 3′ poly(A) tail] with eukaryotic initiation factor 4G (eIF4G) as a bridging scaffold, which in turn interacts with eIF4E (bound to the 5′ cap) in order to facilitate translation (20). PABPC1 also plays an important role in translation by interacting directly with translation initiation factors such as PAIP1 (a translational stimulator) and PAIP2A and PAIP2B (21–23) (reviewed in reference 24). Lastly, PABPC1 also plays an important role in RNA homeostasis, in that it protects the 3′-poly(A) tail until proper signals cause it to dissociate and allow deadenylases (e.g., CAF1-CCR4-NOT complex, PAN2-PAN3 complex) to initiate the degradation of the mRNA.

PABPs have discernible domain architectures that are largely conserved across all eukaryotic organisms studied to date. The cytoplasmic PABPs, such as PABPC1, have four RNA recognition motifs (RRM I to IV) near the N terminus and a C-terminal PABPC or MLLE (“mademoiselle”) domain, as those amino acids (MLLE) are encoded as part of the peptide recognition sequence of this domain (17, 25). RRM I and II have been shown to interact specifically with poly(A) sequences, and RRM III and IV interact nonspecifically with RNA (26). Interestingly, these distinct RRMs are evolutionarily conserved across species, indicating that they have likely evolved for different functions and protein-protein interactions. The PABC/MLLE domain is responsible for interacting with other proteins that contain a PAM2 motif, such as PAIP1, PAIP2A, PAIP2B, ataxin-2, LARP4, and others (21). Nuclear PABPs, such as PABPN1, have only one RRM and a C-terminal portion enriched in Arg residues (17). Structural information with respect to the RRM domains in complex with RNA and some binding partners and a structure of the PABC/MLLE domain are known, but a full-length protein structure of PABP has not yet been solved due to technical constraints (27–30).

Little has been experimentally discovered about PABPs in *Plasmodium* species. However, previous studies of *Plasmodium falciparum* have identified and bioinformatically characterized one PABP (PfPABP) that has the characteristics of a cytoplasmic PABP (four RRMs and a PABC/MLLE domain), but with additional sequences inserted between RRM III and RRM IV as is common for *Plasmodium* proteins. In this work, no other PABP was identified, perhaps because single-celled eukaryotes typically encode only one PABP (31, 32). When the binding activity of PfPABP was measured using recombinant protein by dot blotting, the results showed that it binds to poly(A) sequences specifically over poly(G) and poly(C) sequences and that it was able to associate with the eIF4G scaffold protein specifically (31). Additionally, our previous work unexpectedly showed that the cytoplasmic PABP localizes on the surface of sporozoites (33). While this might have been an artifact of the labeling procedure, recent evidence has also shown that GAPDH (glyceraldehyde-3-phosphate dehydrogenase) is also an RNA-binding protein and is found on the surface of sporozoites in *P. falciparum* (34). Taking the data together, we were intrigued by that fact that *Plasmodium* would choose to position RNA-binding proteins on its surface in the sporozoite stage. Finally, our recent bioinformatic survey of *Plasmodium* RNA-binding proteins has identified three possible PABPs in *Plasmodium* species, including *P. falciparum* and *P. yoelii* (35). We noted that PyPABP1 has characteristics similar to those of cytoplasmic PABP, while what we had previously termed PyPABP3 (and now call PyPABP2) has characteristics similar to those of the nuclear PABP.

In this study, we demonstrated that PyPABP1 and PyPABP2 are in fact cytoplasmic and nuclear PABPs of *P. yoelii*, respectively. *Plasmodium*, although a single-celled eukaryote, resembles metazoans more than other single-celled species in this regard. Using recombinant, full-length PyPABP1 and PyPABP2 proteins, we demonstrated that both proteins bind specifically to poly(A) sequences. Transmission electron microscopy (TEM) of recombinant PyPABP1 showed that it forms chains even upon RNase treatment. Lastly, specific polyclonal antibodies (Abs) showed that these proteins are expressed and localize to the cytosol and nucleus in most stages and, excitingly, that PyPABP1 is in fact localized nearly entirely to the surface of salivary gland sporozoites and is deposited in trails upon parasite gliding on a substrate. Taking the data together, we provide experimental evidence for the function, structure, and localization of these proteins in the nucleus, cytosol, and surface of the malaria parasite.

## RESULTS

### PyPABP1 and PyPABP2 have biochemical and domain characteristics of poly(A)-binding proteins.

A previous study in which various bioinformatic methods were used to search the genomes of several *Plasmodium* species to identify RNA-binding proteins identified three possible poly(A)-binding proteins (PABP) in *Plasmodium yoelii*, which we here call PyPABP1 (PY17X_1441700), PyPABP2 (PY17X_0828100), and (representing a putative PABP) PyPABP3 (PY17X_1129700) (35). We began characterizing these proteins by first analyzing their amino acid sequences to find predicted domains using BLASTp. PyPABP1 has four predicted RNA recognition motifs (RRMs) within 500 amino acids (aa) of the N terminus and a conserved C-terminal domain, known as the PABPC or MLLE domain, which has been known to bind factors that regulate the function of PABP (25). Studies of PABPs in yeast have shown that PABPs with this domain architecture are localized in the cytosol (36, 37). PyPABP2 has one predicted RRM, has an overrepresentation of Arg residues C terminal of its RRM, does not have a proline/glutamine-rich region, and thus has the same domain architecture as and a sequence similar (51% identity) to that of *Homo sapiens* PABPN1, which is a nuclearly localized PABP (38). PyPABP3 has two predicted RRM domains within 250 amino acids of the N terminus; however, this protein does not have a domain architecture similar to any known PABPs from other model organisms and thus we did not pursue it for further experimentation. On the basis of studies of the human and yeast PABPs and our domain predictions, we anticipate PyPABP1 to be localized in the cytoplasm and PyPABP2 to be localized in the nucleus. The arrangement of PyPABP1 and PyPABP2 would match that which is present in model eukaryotes, with PABP2 binding mRNAs in the nucleus and handing them off to PABP1 during nuclear export.

**FIG 1:**
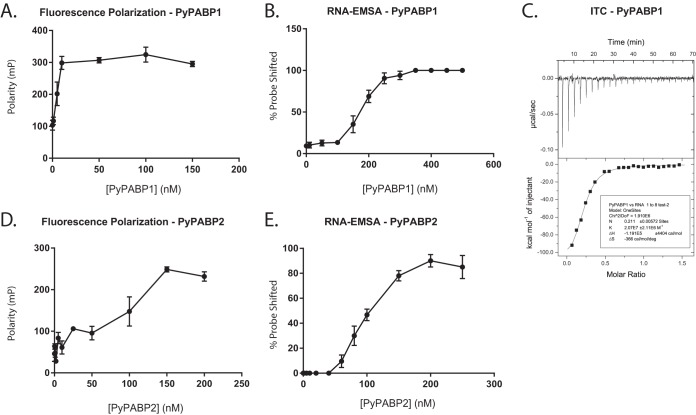
PyPABP1 and PyPABP2 bind specifically to polyadenylated RNA. (A and D) A fluorescence polarization assay using a fluorescein-labeled 25-mer poly(A) probe was used to measure binding of (A) PyPABP1 and (D) PyPABP2 in biological triplicate. (B and E) RNA-EMSA was used to measure binding of (B) PyPABP1 and (E) PyPABP2 using a biotin-labeled 25-mer poly(A) probe in biological triplicate. (C) Isothermal titration calorimetry (ITC) was also performed in biological duplicate on PyPABP1 as a third measure of its binding affinity.

To biochemically characterize these proteins, we investigated their ability to bind polyadenylated RNA using complementary fluorescence polarization (FP) and RNA electromobility shift assay (RNA-EMSA) approaches. Recombinant full-length PyPABP1 and PyPABP2 were extensively purified (see Fig. 1 in the supplemental material) and were allowed to bind with a 25-mer poly(A) probe in the presence of nonspecific competitor RNA. Both the RNA-EMSAs and the FP assays showed that PyPABP1 binds to poly(A) sequences strongly and does so with apparent dissociation constant (*K*_*d*_)_app_ values of 185 nM and 8 nM, respectively (Fig. 1A and B; a representative RNA-EMSA blot is provided in Fig. S2). The (Kd)_app_ derived from the fluorescence polarization assay matches what has been observed for cytosolic PABPs in other eukaryotes (17). However, the binding affinity as measured by RNA-EMSA, although strong, was consistently 2 orders of magnitude lower than that measured by FP. While both assays are established and robust means to determine the binding affinity of protein/nucleic acid interactions, FP measures the rate of molecular tumbling in solution, whereas RNA-EMSA provides a snapshot of these interactions due to caging effects of the gel matrix. Because of this discrepancy between assays, isothermal titration calorimetry (ITC) was used as a third method to determine the affinity of PyPABP1 for poly(A) RNA (Fig. 1C). ITC provides a direct measurement of the *K*_*d*_ of this interaction in solution. When ITC was conducted in biological duplicate with independent protein preparations, the *K*_*d*_ values for PyPABP1 for a 25-mer of poly(A) RNA were 33 and 48 nM (Fig. 1C). Those values are consistent with the results of FP measurements and the published binding affinity values for other PABPs of model eukaryotes.

In the case of PyPABP2, both the FP assays and RNA-EMSAs showed that PyPABP2 binds to poly(A) sequences with nearly identical (*K*_*d*_)_app_ values of 104 nM and 108 nM, respectively (Fig. 1D and E). Moreover, the binding properties of PyPABP1 and PyPABP2 determined by FP were essentially the same in the presence (Fig. S3) and absence (Fig. 1) of excess bovine serum albumin (BSA), as well as in the presence of excess nonspecific RNA (Fig. 1 and S3), indicating that the interaction is specific. These independent assays verify that PyPABP1 and PyPABP2 bind specifically to poly(A) sequences and further demonstrate that these proteins are bona fide poly(A)-binding proteins (PABPs).

### Purified PyPABP1 forms homopolymeric chains.

**FIG 2:**
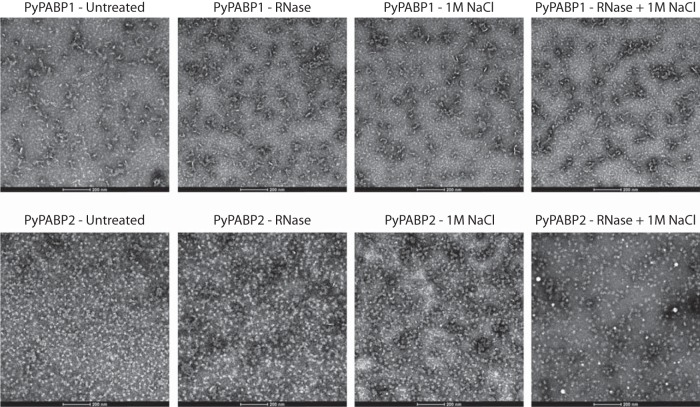
PyPABP1 is sufficient to form chains. Recombinant PyPABP1 and PyPABP2 were left untreated or were treated with RNase to digest accessible RNA, with 1 M NaCl to compete with the RNA/PABP interaction, or with both RNase and high levels of salt. Samples were applied to glow-discharged EM grids and negative stained with PTA. When viewed by negative-stain TEM at ×49,000 magnification, chains of variable lengths were observed for PyPABP1 under all conditions, whereas PyPABP2 remained globular. Scale bar, 200 nm.

As limited structural information is available about full-length PABPs, we used transmission electron microscopy (TEM) to assess the tertiary structure and possible quaternary structure of PyPABP1 and PyPABP2. Highly purified recombinant PyPABP1 or PyPABP2 (0.1 mg/ml; Fig. S1) was applied to carbon-coated grids and negative stained. Micrographs clearly show that PyPABP1 formed chains of different lengths whereas, as anticipated, PyPABP2 formed globular proteins approximately 15 to 17 nm in diameter (Fig. 2). Previous studies have shown that in the presence of RNA, PABPs can cooperatively bind through a proline/glutamine-rich region near the C terminus, which is present on PyPABP1 (39, 40). We hypothesized that PyPABP1 may form chains by interacting with RNA derived from *Escherichia coli*. To test whether chain formation was RNA dependent, purified PyPABP1 was treated with RNase I for 2 h at room temperature (RT) in the presence or absence of 1 M NaCl or was treated with 1 M NaCl without RNase treatment and reanalyzed by negative-stain TEM (Fig. 2). None of these treatments had an effect on chain formation with PyPABP1 or on the globular structure of PyPABP2, and thus PyPABP1 alone either is sufficient to form chains or binds and protects RNA from digestion despite long incubation times in solution conditions that should promote RNA exposure and release. In order to further determine if recombinant PyPABP1 could form homopolymeric chains, analysis of PyPABP1 by size exclusion chromatography confirmed that recombinant PyPABP1 was forming multimers in solution, as it eluted prior to rabbit IgG standards (150 kDa) (Fig. S4). From these data, we conclude that *Plasmodium* may preassemble PABP1 chains for binding to polyadenylated mRNAs.

10.1128/mSphere.00435-17.1 FIG S1  Recombinant PyPABP1 and PyPABP2 are soluble in *E. coli* and are effective antigens for antibody production. Recombinant PyPABP1 and PyPABP2 were expressed in *E. coli*, purified to >95%, and used to produce rabbit polyclonal antibodies. (Top left) Recombinant PyPABP1 was expressed in *E. coli* with approximately 60% solubility. PyPABP1 was purified using Ni-NTA resin via a C-terminal 6×His tag, followed by cation exchange chromatography. (Bottom left) Likewise, recombinant PyPABP2 was expressed in *E. coli* with approximately 80% solubility. PyPABP2 was purified using glutathione agarose resin via a GST tag on the N terminus followed by cation exchange chromatography. (Top right and bottom right) Rabbit polyclonal antibodies were produced using recombinant PyPABP1 and PyPABP2. Preimmune serum was collected preinjection, and the production of specific antibodies was analyzed by Western blotting of membranes containing recombinant protein and *P. yoelii* 17XNL parasite lysates from asexual blood stages, gametocytes, or sporozoites. Download FIG S1, PDF file, 0.1 MB. Copyright © 2018 Minns et al. 2018 Minns et al. This content is distributed under the terms of the Creative Commons Attribution 4.0 International license.

10.1128/mSphere.00435-17.2 FIG S2  Representative RNA-EMSA images demonstrate specific poly(A) RNA binding by PyPABP1 and PyPABP2. One gel image (representative of three) from RNA-EMSAs for PyPABP1 (left) and one for PyPABP2 (right) are shown. Download FIG S2, PDF file, 0.03 MB. Copyright © 2018 Minns et al. 2018 Minns et al. This content is distributed under the terms of the Creative Commons Attribution 4.0 International license.

10.1128/mSphere.00435-17.3 FIG S3  PyPABP1 and PyPABP2 bind specifically to poly(A) RNA sequences. To additionally show that PyPABP1 and PyPABP2 bind specifically to poly(A) sequences, fluorescence polarization assays were performed using recombinant (A) PyPABP1 and (B) PyPABP2 and a fluorescein-labeled 25-mer poly(A) RNA probe and were analyzed as described for Fig. 1 but in the presence of 0.1 mg/ml BSA. No significant difference in binding properties was observed. Download FIG S3, PDF file, 0.8 MB. Copyright © 2018 Minns et al. 2018 Minns et al. This content is distributed under the terms of the Creative Commons Attribution 4.0 International license.

10.1128/mSphere.00435-17.4 FIG S4  Recombinant PyPABP1 forms multimers in solution. Purified, recombinant PyPABP1 was subjected to size exclusion chromatography using an S300 column. Size standards (BSA, 66 kDa; rabbit IgG, 150 kDa) and the void volume were used to interpolate the PyPABP1 complex size as being multimeric. Download FIG S4, PDF file, 2.5 MB. Copyright © 2018 Minns et al. 2018 Minns et al. This content is distributed under the terms of the Creative Commons Attribution 4.0 International license.

### PyPABP1 is localized in the cytosol in blood stage parasites but localizes to the surface of sporozoites.

**FIG 3:**
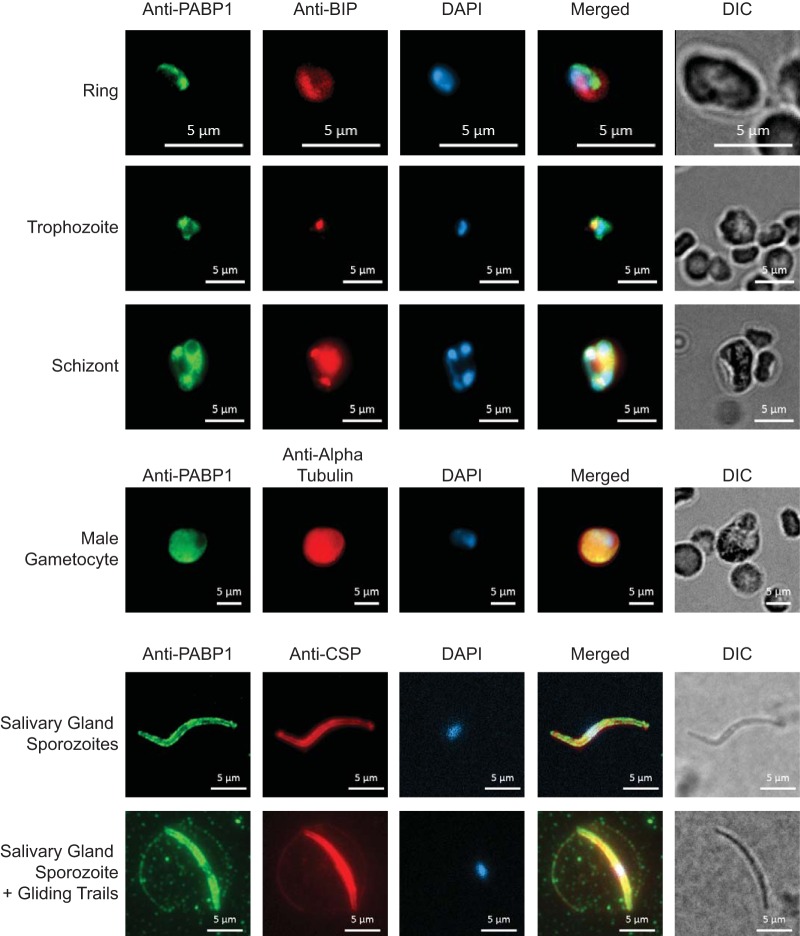
PyPABP1 is cytosolic but is found on the surface of sporozoites. Indirect immunofluorescence assay (IFA) images show that PyPABP1 localizes in the cytosol and adjacent to the nucleus in blood stage rings, trophozoites, and schizonts (top) and in male gametocytes (middle). However, PyPABP1 localizes to the plasma membrane of salivary gland sporozoites and is deposited in trails during gliding (bottom). In all stages, PyPABP1 expression and localization were visualized using a specific polyclonal antibody against recombinant full-length PyPABP1 and a species-specific Alexa Fluor-conjugated secondary antibody, and DAPI was used to visualize the nucleus. Parasites were counterstained with (A) an anti-PvBIP antibody (asexual blood stages) to visualize the endoplasmic reticulum, (B) an anti-alpha tubulin II antibody (male gametocytes) to visualize the cytosol, or (C) an anti-PyCSP (clone 2F6) antibody (salivary gland sporozoites) to visualize the plasma membrane and gliding trails.

On the basis of the domain architecture predicted from model eukaryotes, PyPABP1 is expected to be a cytosolic poly(A)-binding protein. To characterize the expression and localization of PyPABP1, we generated rabbit polyclonal antibodies against the full-length, soluble protein. PyPABP1 antibodies were highly reactive to both recombinant protein (glutathione *S*-transferase [GST]-PyPABP1; 123 kDa) and a protein species that migrates at a slightly lower rate than that predicted for the molecular mass for the native protein (96 kDa) in parasite lysates from asexual blood stages, gametocytes, and sporozoites (Fig. S1). Due to the presence of the highly charged patches of amino acids found in PyPABP1, slower mobility of this protein as observed by SDS-PAGE is appropriate and matches that seen with other proteins. Next, we performed indirect immunofluorescence assays (IFAs) on asexual blood, male gametocyte, and sporozoite stage parasites (Fig. 3). As anticipated, PyPABP1 is cytosolically localized in asexual ring, trophozoite, and schizont stage parasites, as well as in male gametocytes (Fig. 3). Moreover, PyPABP1 is also found adjacent to the nucleus, which is best demonstrated by the image of the asexual schizont stage parasites (Fig. 3). This likely reflects the fact that, as with model eukaryotes, PyPABP1 is poised to receive mRNAs exported from the nucleus. In stark contrast, we found that PyPABP1 is instead also localized to the surface of salivary gland sporozoites and is deposited in trails when sporozoites are allowed to glide on a substrate (Fig. 3). These data directly support our previous proteomic labeling experiments performed with *P. falciparum* salivary gland sporozoites that robustly showed that PfPABP1 is surface exposed (33). Moreover, this is the third RNA-binding protein known to localize on the surface of sporozoites, as tRip and GAPDH were also recently found to localize on the surface as well (34, 41). Taking the results together, we found that PyPABP1 is localized in the cytosol and nuclear periphery throughout the sexual and asexual blood stages but becomes surface localized and is shed by salivary gland sporozoites during gliding motility. This raises unaddressed issues of why sporozoites would place RNA-binding proteins on their surface and what function may they perform here.

**FIG 4:**
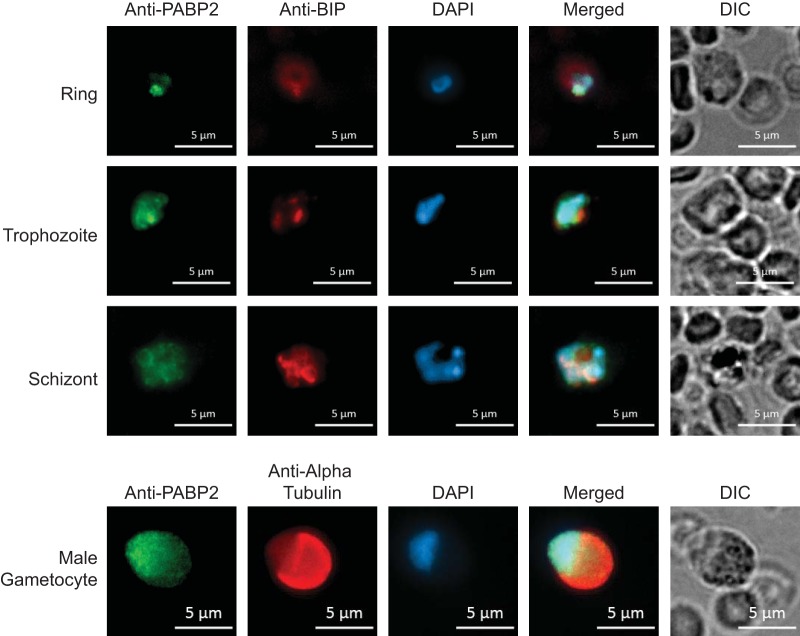
PyPABP2 is largely found in the nucleus. Indirect immunofluorescence assay (IFA) images show that PyPABP2 localizes to the nucleus in blood stage rings, trophozoites, and schizonts (top) and in male gametocytes (middle). However, it is partially cytosolic in male gametocytes. In these stages, PyPABP2 expression and localization were visualized using a specific polyclonal antibody against recombinant full-length PyPABP2 and a species-specific Alexa Fluor-conjugated secondary antibody, and DAPI was used to visualize the nucleus. Parasites were counterstained with (A) an anti-PvBIP antibody (asexual blood stages) to visualize the endoplasmic reticulum or (B) an anti-alpha tubulin II antibody (male gametocytes) to visualize the cytosol. No specific staining was observed for sporozoites, consistent with the higher background observed by Western blotting with sporozoite lysates.

To characterize PyPABP2 expression and localization, we again performed IFAs using a polyclonal antibody derived from recombinant, full-length PyPABP2. As with PyPABP1, Western blotting of recombinant (52 kDa) and native (23 kDa) PyPABP2 confirmed the specificity of the antibody in asexual blood stages and gametocytes (Fig. S1). However, strongly reactive bands in addition to the expected species were observed with lysates from sporozoites (Fig. S1). On the basis of domain architecture predictions, PyPABP2 was expected to be localized within and near the nucleus. PyPABP2 largely colocalizes with DAPI (4′,6-diamidino-2-phenylindole) in sexual and asexual blood stages, demonstrating that it is associating with the nucleus (Fig. 4). We also observed that a portion of PyPABP2 also surrounds the DAPI signal, suggesting that PyPABP2 is nucleus adjacent and as in model eukaryotes, may be exported from the nucleus with bound mRNAs to hand them off to the cytosolic PyPABP1 (Fig. 4). Unexpectedly, while we observed that most of the PyPABP2 was present in the nucleus of male gametocytes, we also found that an appreciable amount of PyPABP2 was cytosolic. Lastly, while interrogation of expression and localization of PyPABP2 in other stages would be of interest, these antisera did not have sufficient specificity for their use with salivary gland sporozoites to determine if this protein also changes its localization in this stage.

## DISCUSSION

Poly(A)-binding proteins are important and tied to many cellular processes, including translation, promotion of RNA polyadenylation, and RNA degradation. In this report, we have provided evidence that *Plasmodium yoelii* utilizes two PABPs, PyPABP1 (PY17X_1441700) and PyPABP2 (PY17X_0828100), which are cytoplasmic and nuclear PABPs, respectively. PyPABP1 has a domain architecture similar to that of cytoplasmic PABPs from model eukaryotes, with four N-terminal RRM domains and a C-terminal MLLE/PABPC domain that are bioinformatically predicted. Our binding assays demonstrate that PyPABP1 binds to poly(A) sequences specifically, with both FP and ITC showing that its high binding affinity for poly(A) RNA matches that seen in model eukaryotes. We also observed that PyPABP2 bound to poly(A) RNA specifically by RNA-EMSA and fluorescence polarization (FP) but did so about 10-fold more weakly. This may reflect differences in how PyPABP1 and PyPABP2 interact with poly(A) RNA in the cell and may facilitate the handoff of poly(A) mRNAs upon nuclear export.

Interestingly, purified, recombinant PyPABP1 forms homopolymeric chains that are resistant to RNase digestion and high-salt treatment. These data were confirmed by observations that PyPABP1 forms multimers in solution by size exclusion chromatography. It is known that cytoplasmic PABPs cooperatively interact through a proline/glutamine-rich region near the C terminus when bound to RNA (39, 40). That the chains persisted even after RNase and high-salt treatment suggests that PyPABP1 may preassemble before interacting with polyadenylated RNA. Alternatively, PyPABP1 may form and retain chains that can strongly protect bound RNA over extended periods of time at physiological temperatures. In stark contrast, PyPABP2 does not contain a proline/glutamine-rich patch typically used for multimerization and does not form chains under any of these conditions.

As anticipated, PyPABP1 localizes in the cytoplasm of blood stage parasites by IFA. Unexpectedly, however, PyPABP1 was found to instead localize to the surface of salivary gland sporozoites and was deposited in trails along with circumsporozoite protein (CSP) during sporozoite gliding on a substrate. Our previous work identified the ortholog of PABP1 in *P. falciparum* as a surface-exposed protein, but that result could have been presumed to be an artifact of the assay, as PfPABP1 is an abundant protein and we reported that a small fraction of the sporozoites had leaky membranes or were dead. However, here we have experimentally validated that it is a bona fide surface-exposed protein. This is now the third RNA-binding protein found on the surface of sporozoites, with combined data supporting this identification derived from mass spectrometric detection, reverse genetics, and immunostaining approaches. Understanding why this form of the parasite employs RNA-binding proteins on its surface may provide additional clues to how it interacts with other parasites and/or the host. Previous work has demonstrated that sporozoites are able to internalize exogenous tRNAs by the activity of the tRip protein (41). The presence of PyPABP1 (and presumably its orthologues in other species) may provide another means to bind extracellular RNA found in exosomes or other structures.

Typically, single-celled eukaryotes have a single, cytoplasmic PABP whereas metazoans have at least two, a nuclear PABP and a cytoplasmic PABP. *Plasmodium* spp. and several other apicomplexans (e.g., *Toxoplasma*, *Hammondia*, *Neospora*) that encode PABP2-like proteins are therefore unusual among single-celled eukaryotes because they have a nuclear PABP as well. *Schizosaccharomyces pombe*, or fission yeast, is another single-celled eukaryote that has a nuclear PABP, which is *Schizosaccharomyces pombe* PAB2 (SpPAB2). Unlike *H. sapiens* PABPN1 (HsPABPN1; the human nuclear PABP), SpPAB2 does not appear to participate in RNA polyadenylation (42). Here we found that PyPABP2 is a nuclear-associated PABP on the basis of its domain architecture, RNA-binding activity, and localization in the parasite. While our IFA micrographs demonstrate that PyPABP2 localizes mostly in the nucleus, some also localizes to a nucleus-adjacent region that may reflect a zone where mRNAs may be handed off to PyPABP1.This is especially evident in male gametocytes, where an appreciable amount of PyPABP2 appears to localize in the cytoplasm. PyPABP2 and SpPAB2 (199 aa and 166 aa, respectively) are closer in size to each other than either is to PABPN2 (306 aa) and may provide clues to the functions of PyPABP2.

Together, these PABPs have been associated with translational repression complexes in *Plasmodium* and are likely to be key regulators as they are for other eukaryotes (5, 13). While we provide characterization of both PyPABP1 and PyPABP2 here using both biochemical and parasitological approaches, further structural and kinetics investigations of these two proteins are warranted and should reveal nuances in their roles in the nucleus and cytoplasm and on the surface.

## MATERIALS AND METHODS

### Recombinant protein expression and purification of PyPABP1.

Full-length PyPABP1 (PY17X_1441700; AA1-835) was PCR amplified using *P. yoelii* 17XNL genomic DNA and Phusion polymerase and primers (sequences provided in Table S1 in the supplemental material) and was then ligated into pCR-Blunt for sequencing. The sequence-verified fragment was excised with NcoI and XhoI and ligated into a modified pET28b+ expression vector (pSL0220) that incorporates a GST tag on the N terminus and a 6×His tag on the C terminus of the expressed protein, which can be cleaved with thrombin and TEV protease, respectively. The recombinant protein was expressed in the Rosetta2 (DE3) pLysS strain of *E. coli* grown in 20 liters of LB broth using a 30-liter fermenter. To produce soluble protein, when the culture achieved an optical density at 600 nm (OD_600_) of 0.6, protein expression was induced by addition of 0.2 mM (final concentration) IPTG (isopropyl-β-d-thiogalactopyranoside) at 21°C for 17 h. The cell pellet was suspended in Low-imidazole buffer (25 mM Tris-Cl [pH 7.5] at room temperature [RT], 500 mM NaCl, 10 mM imidazole, 1 mM dithiothreitol [DTT], 1 mM benzamidine, and 10% glycerol) and lysed by 10 rounds of sonication, with each round consisting of 20 pulses at 30% amplitude and 50% duty cycle (model 450 Branson Digital Sonifier; Disruptor horn). The crude extract was spun at 15,500 × *g* for 10 min at 4°C. The supernatant was incubated with 5 ml of nickel-nitrilotriacetic acid (Ni-NTA) resin (Thermo Scientific catalog no. 88223) equilibrated in Low-imidazole buffer on a nutator for 1 h at 4°C. The resin was washed with 4 column volumes (CV) of Mid-imidazole buffer (25 mM Tris-Cl [pH 7.5] at RT, 500 mM NaCl, 50 mM imidazole, 1 mM DTT, 1 mM benzamidine, and 10% glycerol) and then eluted using a linear gradient of 0% to 100% buffer B over 15 CV (buffer A, 25 mM Tris-Cl [pH 7.5] at RT, 500 mM NaCl, 10 mM imidazole, 1 mM DTT, 1 mM benzamidine, and 10% glycerol; buffer B, 25 mM Tris-Cl [pH 7.5] at RT, 500 mM NaCl, 300 mM imidazole, 1 mM DTT, 1 mM benzamidine, and 10% glycerol). The pooled elution fractions containing the recombinant protein were dialyzed into a mixture of 10 mM HEPES (pH 6.74) at RT, 100 mM NaCl, 1 mM DTT, 1 mM benzamidine, and 10% glycerol. Next, the protein was purified further using cation exchange chromatography using a 20-ml bed volume of SP-Sepharose resin that was equilibrated in buffer A (10 mM HEPES [pH 6.74] at RT, 100 mM NaCl, 1 mM DTT, 1 mM benzamidine, and 10% glycerol). The column was then washed using 3 CV buffer A and eluted using a linear gradient of 0% to 50% buffer B (10 mM HEPES [pH 6.74] at RT, 1,000 mM NaCl, 1 mM DTT, 1 mM benzamidine, and 10% glycerol) over 20 CV. The elution fractions containing the recombinant protein were pooled and exhaustively dialyzed into a mixture of 20 mM MES (morpholineethanesulfonic acid) (pH 6.0) at RT, 100 mM NaCl, 100 mM MgCl_2_, 1 mM DTT, and 10% glycerol. Lastly, the purified protein was concentrated to 10 to 12 mg/ml using Amicon Ultra Centrifugal Filters (Fisher Scientific catalog no. UFC9-003-08).

10.1128/mSphere.00435-17.5 TABLE S1  Oligonucleotides used in this study. Download TABLE S1, XLSX file, 0.01 MB. Copyright © 2018 Minns et al. 2018 Minns et al. This content is distributed under the terms of the Creative Commons Attribution 4.0 International license.

### Recombinant protein expression and purification of PyPABP2.

Full-length PyPABP2 (PY17X_0828100; AA1-199) was synthesized (sequence provided in Table S2) and ligated into pUC57, and the sequence was verified by Genewiz (South Plainfield, NJ). Due to its small size, recodonization of the coding sequence was practical in order to improve expression in *E. coli*, as has been seen for other *Plasmodium* proteins. The sequence was excised using NcoI and XhoI and ligated into the pSL0220 expression vector (described above). The recombinant protein was expressed in the CodonPlus(DE3) strain of *E. coli*, which also carried a plasmid that expressed sigma32 (I54N) to initiate a heat shock-like response to aid the solubility of PyPABP2 (43). Bacteria were grown in 20 liters of LB broth using a 30-liter fermenter, and upon achievement of an OD_600_ of 0.4, protein expression was induced with 0.02% (wt/vol) arabinose to initiate the heat shock response, followed by 0.2 mM IPTG to induce PyPABP2 expression at 21°C for 17 h. The cell pellet was suspended in GST lysis buffer (50 mM Tris-Cl [pH 8.0] at RT, 150 mM NaCl, 1 mM DTT, 1 mM benzamidine, and 10% glycerol) and lysed by 4 rounds of sonication, with each round consisting of 30 cycles at 50% amplitude and 50% duty cycle (model 450 Branson Digital Sonifier; Disruptor horn). The supernatant was incubated with 3 ml of glutathione agarose resin (Thermo Scientific catalog no. PI16101) equilibrated in GST lysis buffer on a nutator for 1 h at 4°C. The resin was washed with 30 ml GST lysis buffer and then eluted using a linear gradient of 0% to 100% buffer B over 20 CV (buffer A, 50 mM Tris-Cl [pH 8.0] at RT, 150 mM NaCl, 1 mM DTT, and 10% glycerol; buffer B, 50 mM Tris-Cl [pH 8.0] at RT, 150 mM NaCl, 30 mM reduced glutathione, 1 mM DTT, and 10% glycerol). The pooled elution fractions containing the recombinant protein were dialyzed into a mixture of 20 mM MES (pH 6.0) at RT, 100 mM NaCl, 1 mM DTT, and 10% glycerol. Next, the protein was purified further using cation exchange chromatography and a 20-ml bed volume of SP-Sepharose resin that was equilibrated in buffer A (20 mM MES [pH 6.0] at RT, 100 mM NaCl, 1 mM DTT, and 10% glycerol). The column was then washed using 3 CV buffer A and then eluted using a linear gradient of 30% to 90% buffer B (20 mM MES [pH 6.0] at RT, 1,000 mM NaCl, 1 mM DTT, and 10% glycerol) over 20 CV. The elution fractions containing the recombinant protein were pooled and exhaustively dialyzed into a mixture of 20 mM Tris (pH 7.5) at RT, 150 mM NaCl, 1 mM DTT, and 10% glycerol. Lastly, the purified protein was concentrated to 10 to 12 mg/ml using Amicon Ultra Centrifugal Filters (Fisher Scientific catalog no. UFC9-003-08).

10.1128/mSphere.00435-17.6 TABLE S2  Synthetic sequence of PyPABP2. Download TABLE S2, XLSX file, 0.01 MB. Copyright © 2018 Minns et al. 2018 Minns et al. This content is distributed under the terms of the Creative Commons Attribution 4.0 International license.

### RNA electromobility shift assay (RNA-EMSA).

Protein/RNA interactions were determined by RNA-EMSA performed using increasing concentrations of recombinant protein (for PyPABP1, 0 nM, 10 nM, 50 nM, 100 nM, 150 nM, 200 nM, 250 nM, 300 nM, 350 nM, 400 nM, 450 nM, and 500 nM; for PyPABP2, 0 nM, 1 nM, 5 nM, 10 nM, 20 nM, 40 nM, 60 nM, 80 nM, 100 nM, 150 nM, 200 nM, and 250 nM) and a constant concentration of a biotinylated 25-mer poly(A) RNA probe (IDT) (for PyPABP1, 0.25 nM; for PyPABP2, 0.5 nM). The reaction mixture was composed of 10 mM Tris-Cl (pH 7.5) at RT, 5 mM MgCl_2_, 55 mM KCl, 2 µg tRNA (Sigma catalog no. R8508) (1 ml), 1 mM DTT, 0.5 U/µl SUPERase-In (Invitrogen; AM2694), and 2.5% (vol/vol) glycerol, to which the probe and then protein were added last. The samples were allowed to reach equilibrium at room temperature for 20 min and were then electrophoresed on a native 6% polyacrylamide gel at 100 V for 1 h in chilled 1× Tris-borate-EDTA (TBE) buffer. The gel was then transferred to a nylon membrane for 1 h at 380 mA in chilled 1× TBE buffer, and the resulting blot was washed, developed, visualized, and quantified using a LightShift chemiluminescent EMSA kit (nucleic acid detection module; Thermo Fisher Scientific catalog no. PI20148), a ChemiDoc XRS+ system, and Image Lab Software 5.0 (BioRad). The (*K*_*d*_)_app_ value was calculated by determining the protein concentration at which there was a 50% shift of the probe from unbound to bound. A total of three biological replicate experiments were conducted, and the means and standard errors of the means (SEM) were calculated.

### Fluorescence polarization.

Protein/RNA interactions were also determined by fluorescence polarization (FP) using increasing concentrations of recombinant protein (for PyPABP1, 0 nM, 1 nM, 5 nM, 10 nM, 50 nM, 100 nM, and 150 nM; for PyPABP2, 0 nM, 0.5 nM, 1 nM, 2 nM, 5 nM, 10 nM, 25 nM, 50 nM, 100 nM, 150 nM, and 200 nM) and a constant concentration (0.76 nM) of a fluorescein-labeled 25-mer poly(A) RNA probe (IDT). The reaction mixture was composed of 10 mM Tris-Cl (pH 7.5) at RT, 5 mM MgCl_2_, 55 mM KCl, 2 µg tRNA (Sigma catalog no. R8508; 1 ml), 1 mM DTT, 0.5 U/µl SUPERase-In (Invitrogen; AM2694), and 2.5% (vol/vol) glycerol, to which the probe and then protein were added last. Additional replicates were run that included 0.1 mg/ml BSA in the reactions, and no significant differences in binding were observed. The samples were allowed to reach equilibrium at room temperature for 20 min. An additional control sample was created that contained no protein or probe in order to subtract background signals. The samples were transferred into a Corning black 96-well plate, and fluorescence readings were measured with a SpectraMax M5 multiplate reader with Max Pro 6.3 software (Molecular Devices). The data generated were first normalized by subtracting the background (the negative-control sample measurement) from values determined for the experimental samples. The perpendicular and parallel measurements of emitted polarized light were used to calculate the fluorescence polarization (mP) value for the each sample using the following equation: mP = 1,000 × [parallel [minus] (*G* × perpendicular)/parallel + (*G* × perpendicular)]. The resulting data were then plotted, and the (*K*_*d*_)_app_ was determined as described above for RNA-EMSA. A total of three biological replicate experiments were conducted, and means and standard errors of the means (SEM) were calculated.

### Isothermal titration calorimetry.

Properties of the interaction of PyPABP1 with poly(A) RNA were determined using isothermal titration calorimetry (ITC) and a MicroCal Auto-iTC_200_ calorimeter (Malvern). ITC measurements were performed using recombinant PyPABP1 and a 25-mer poly(A) RNA oligonucleotide, both in buffer containing 10 mM HEPES (pH 7.5), 100 mM KCl, 5 mM MgCl_2_, 1 mM 2-mercaptoethanol, and 5% glycerol. The syringe contained the RNA oligonucleotide at a concentration of 8 µM. A reference run was performed with RNA titrated at 1.5 µl per injection (with 25 injections total) into the cell containing 400 µl of buffer alone at 25°C. Experimental measurements of PyPABP1 were performed with 400 µl of PyPABP1 at a concentration of 1 µM in the cell using parameters identical to those used for the reference run.

### Experimental animals and parasite line.

Six-to-eight-week-old female Swiss Webster (SW) mice were obtained from Envigo (formerly Harlan) and were used for experimental infections and transmission to mosquitoes. *Plasmodium yoelii* strain 17XNL parasites were cycled between mice and *Anopheles stephensi* mosquitoes (originally obtained from the Center for Infectious Disease Research, Seattle, WA) reared at 24°C in 70% humidity. All animal care strictly followed the Association for Assessment and Accreditation of Laboratory Animal Care (AAALAC) guidelines and was approved by the Pennsylvania State University Institutional Animal Care and Use Committee (IACUC number 42678-1).

### Indirect immunofluorescence assays (IFA).

Expression and localization of PyPABP1 and PyPABP2 in asexual blood stages, the male sexual stage, and salivary gland sporozoites were visualized by an indirect immunofluorescence assay. All parasite samples were produced as described previously (44, 45) and were stained at room temperature with the following antibodies: rabbit anti-PyPABP1 (Pocono Rabbit Farm and Laboratory; custom polyclonal antibody [PAb]) (1:500 to 1:1,000), rabbit anti-PyPABP2 (Pocono Rabbit Farm and Laboratory; custom PAb) (1:500 to 1:1,000), mouse anti-PvBiP (1:1,000) (46), mouse anti-alpha tubulin (B-5-1-2) (Sigma-Aldrich catalog no. T5168 Research Resource Identifier [RRID]:AB_477579) (1:1,000), and mouse anti-CSP (clone 2F6; 23356439) (1:1,000). Secondary antibodies were Alexa Fluor conjugated (AF488 and AF594) and specific to rabbit or mouse IgG (Thermo Fisher Scientific catalog no. A-11001, A-11005, A11008, and A-11012) (1:1,000). Nucleic acids were stained with 4′,6-diamidino-2-phenylindole (DAPI; 1 ng/ml). Samples were treated with VectaShield antifade reagent (Vector Laboratories, VWR; catalog no. 101098-048), overlaid with a coverslip, and sealed with nail polish. Fluorescent and differential inference contrast (DIC) images were taken using a Zeiss fluorescence/phase-contrast microscope (Zeiss Axioscope A1 with 8-bit AxioCam ICc1 camera) using a 100× oil objective and processed by Zen imaging software.

### Negative-stain transmission electron microscopy.

Recombinant PyPABP1 and PyPABP2 were purified to near-homogeneity and then left untreated or treated with 20 µg/ml (final concentration) bovine pancreatic RNase for 2 h at room temperature or treated by inclusion of NaCl to reach a 1 M final concentration or treated with the combination of RNase and high salt. Subsequently, 3 µl of purified sample was applied to a freshly glow-discharged continuous carbon grid (Electron Microscopy Sciences catalog no. G400-Cu). The loaded grid was washed with buffer (20 mM MES [pH 6] at RT, 100 mM NaCl, 100 mM MgCl_2_), blotted, and stained with 3 µl 1% (wt/vol) phosphotungstic acid (PTA). The grids were imaged using a FEI Tecnai G2 Spirit BioTwin transmission electron microscope at 120 kV accelerating voltage and magnification at ×49,000 in the Penn State Microscopy and Cytometry Facility—University Park, PA.
