# Supplementary material for: Nuclear, Cytosolic, and Surface-Localized Poly(A)-Binding Proteins of Plasmodium yoelii
Source: mSphere. 2018 Jan 10;3(1):e00435-17. doi: 10.1128/mSphere.00435-17 (PMC5760745; doi:10.1128/mSphere.00435-17)
Supplement: FIG S1 [file sph001182447sf1.pdf]

Figure S1

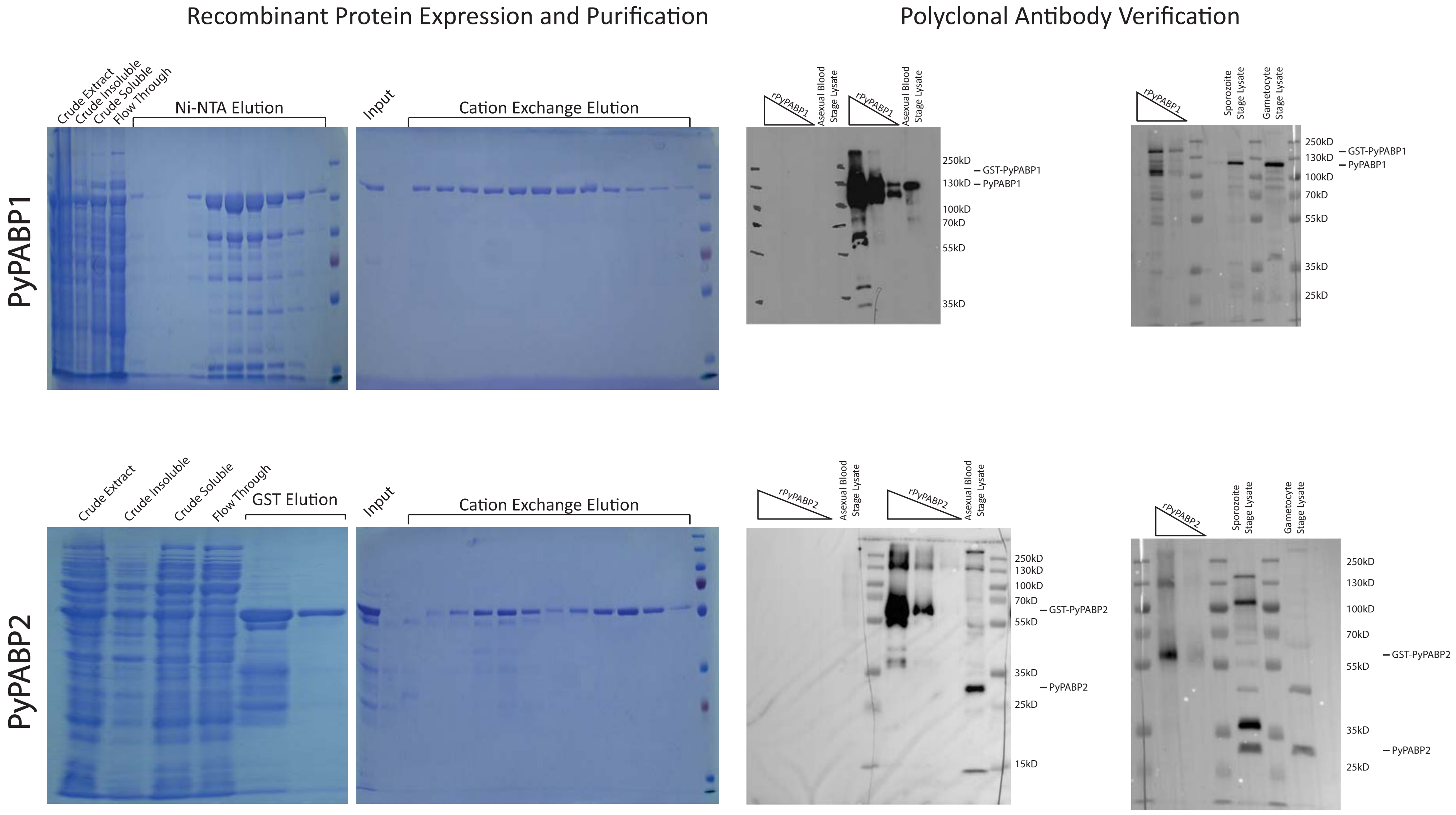
Recombinant Protein Expression and Purification
Polyclonal Antibody Verification
PyPABP1
Crude Extract
Crude Insoluble
Crude Soluble
Flow Through
Ni-NTA Elution
Input
Cation Exchange Elution
rPyPABP1
Asexual Blood Stage Lysate
rPyPABP1
Asexual Blood Stage Lysate
250kD
130kD
100kD
70kD
55kD
35kD
GST-PyPABP1
PyPABP1
rPyPABP1
Sporozoite Stage Lysate
Gametocyte Stage Lysate
250kD
130kD
100kD
70kD
55kD
35kD
25kD
GST-PyPABP1
PyPABP1
PyPABP2
Crude Extract
Crude Insoluble
Crude Soluble
Flow Through
GST Elution
Input
Cation Exchange Elution
rPyPABP2
Asexual Blood Stage Lysate
rPyPABP2
Asexual Blood Stage Lysate
250kD
130kD
100kD
70kD
55kD
35kD
25kD
15kD
GST-PyPABP2
PyPABP2
rPyPABP2
Sporozoite Stage Lysate
Gametocyte Stage Lysate
250kD
130kD
100kD
70kD
55kD
35kD
25kD
GST-PyPABP2
PyPABP2
