# Supplementary figures and images for: Nuclear, Cytosolic, and Surface-Localized Poly(A)-Binding Proteins of Plasmodium yoelii
Source: mSphere. 2018 Jan 10;3(1):e00435-17. doi: 10.1128/mSphere.00435-17 (PMC5760745; doi:10.1128/mSphere.00435-17)

# Figure S2

A.

RNA-EMSA Blot for PyPABP1

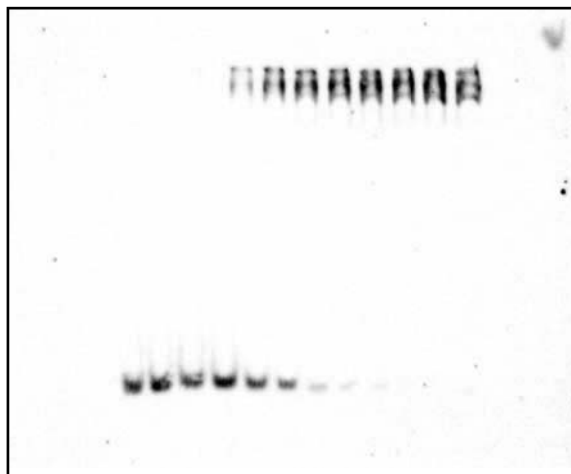

B.

RNA-EMSA Blot for PyPABP2

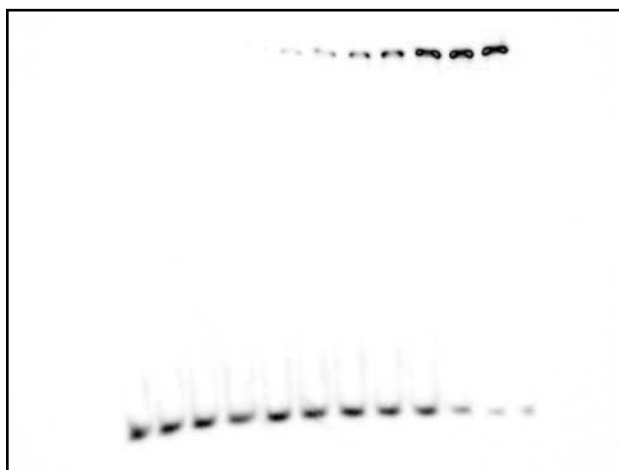

Supplement: FIG S2 [file sph001182447sf2.pdf]

Figure S3

A.

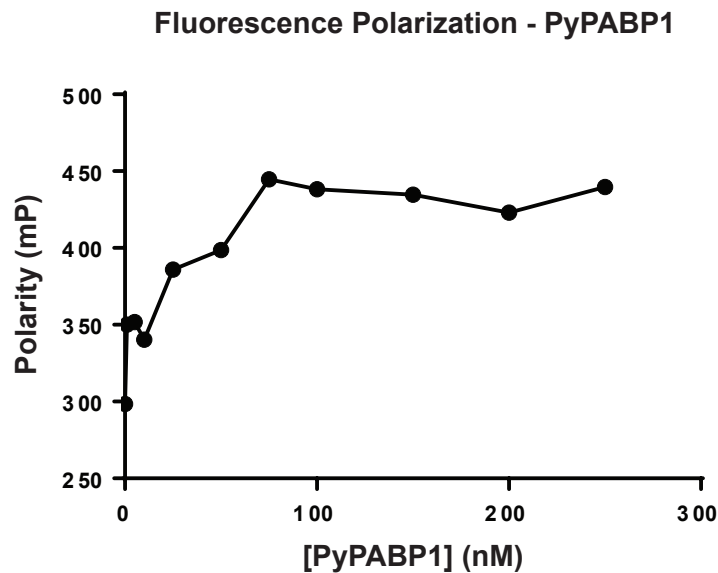

B.

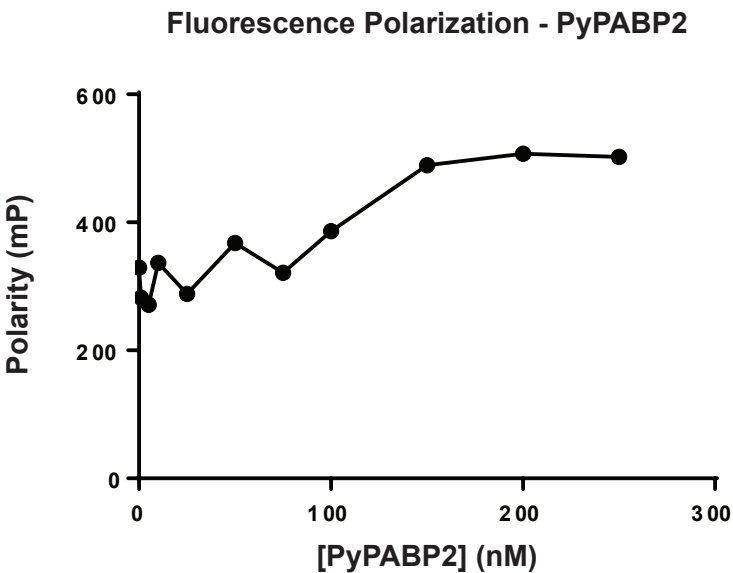

Supplement: FIG S3 [file sph001182447sf3.pdf]

# Figure S4

A.

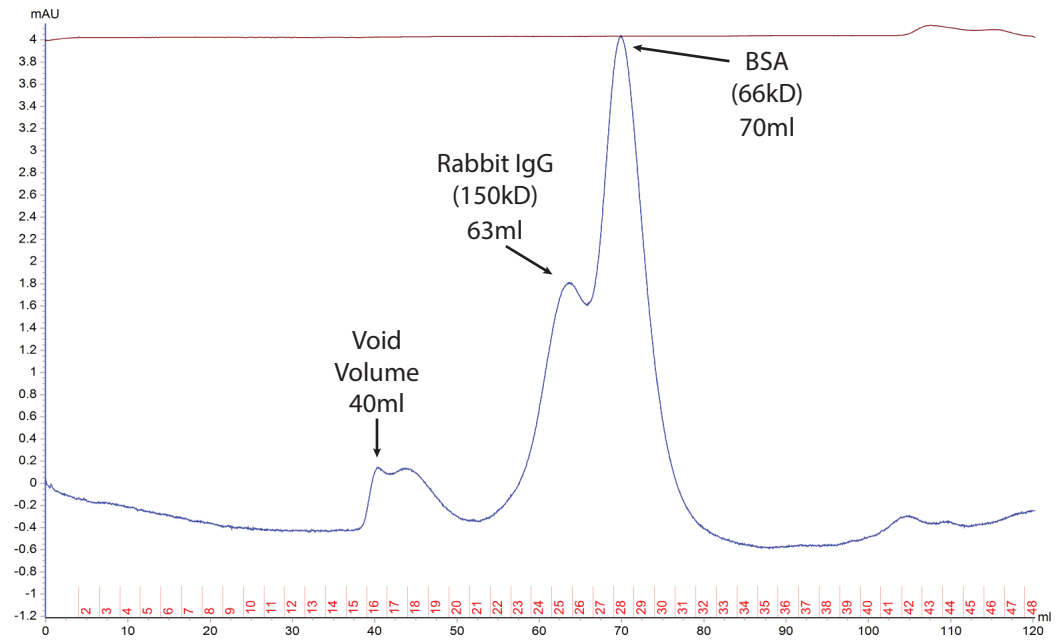

B.

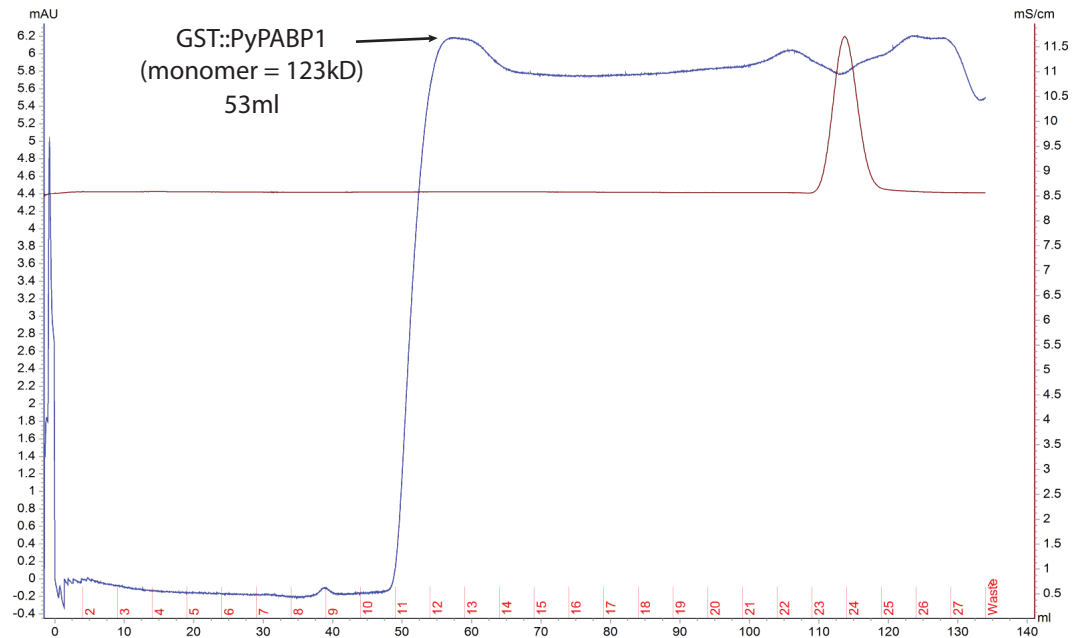

C.

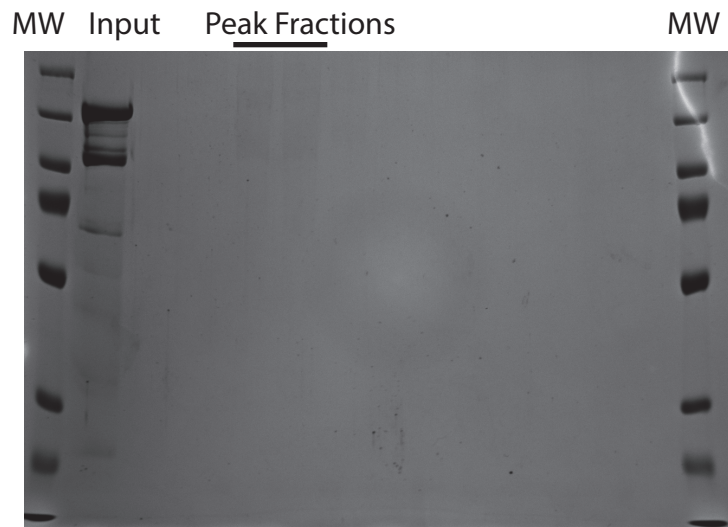

Supplement: FIG S4 [file sph001182447sf4.pdf]
